# Can you feel it? Altered affective touch perception in a transdiagnostic sample of female adolescents with nonsuicidal self-injury

**DOI:** 10.1038/s41398-025-03759-9

**Published:** 2025-11-24

**Authors:** Maria Zetterqvist, Michaela Olofsson, Håkan Olausson, Martin P. Paulus, Markus Heilig, Irene Perini

**Affiliations:** 1 Center for Social and Affective Neuroscience, Department of Biomedical and Clinical Sciences, Linköping university, Linköping, Sweden; 2 Clinical Department of Child and Adolescent Psychiatry in Linköping, Region Östergötland, Linköping, Sweden; 3 Center for Medical Image Science and Visualization, Linköping university, Linköping, Sweden; 4 Department of Psychiatry, University of California, San Diego, La Jolla, USA; 5 Laureate Institute for Brain Research, Tulsa, OK USA

**Keywords:** Neuroscience, Human behaviour

## Abstract

Nonsuicidal self-injury (NSSI), intentionally inflicting harm to one’s body without suicidal intent, is a significant mental health problem in adolescents. Biological mechanisms underlying NSSI are still not well understood but there is some support for altered pain sensitivity. Here we explored the processing of affective touch, an interoceptive modality which has not been explored previously in NSSI. Using functional magnetic resonance imaging, we examined how adolescents with NSSI (*N* = 27), compared to controls (*N* = 26), responded to stroking touch. In the scanner, participants rated the pleasantness and intensity of slow and fast stroking touch on their forearm. A small-volume correction analysis focusing on bilateral insula was performed in addition to a whole-brain analysis. For both approaches and for behavioral ratings, 2×2 factorial ANOVAs were performed. Social touch was also assessed via the Social Touch Questionnaire. Compared to controls, the NSSI group had lower pleasantness ratings, for both slow and fast stroking touch, and higher activity during touch in the insular cortex, a key structure in interoceptive processing. In addition, adolescents with NSSI self-reported avoidance toward social touch. Results suggest that this transdiagnostic clinical sample of adolescent females with NSSI exhibits avoidance to social touch, lower hedonic appreciation of dynamic, innocuous touch, and heightened insular activity in response to touch, potentially linked to altered interoceptive processing.

## Introduction

Nonsuicidal self-injury (NSSI), the deliberate and self-inflicted damage to the body performed without suicidal intent [1], is a significant mental health problem in adolescents. The lifetime prevalence of NSSI in adolescents is around 17–18% [2, 3]. With even higher rates in clinical samples [4], the behavior constitutes a major concern in clinical practice, for example by increasing the risk of suicide [5]. In the latest fifth edition text-revision version of the Diagnostic and Statistical Manual of Mental Disorders (DSM; [6]), diagnostic codes for NSSI were added in Section II, Other conditions that may be a focus of clinical attention, hereby emphasizing its potential to influence prognosis and treatment. In clinical samples, NSSI is comorbid with several mental disorders [7] and NSSI can be conceptualized as a transdiagnostic behavior [8]. Difficulties with regulating emotions are consistently associated with NSSI [9] and NSSI is most often used to reduce strong negative emotions. Other common functions are to self-punish and/or to generate feelings when feeling numb [10]. In addition, interpersonal factors, such as perceived social support and rejection, can also play an important role in the onset, maintenance and cessation of NSSI [10–12]. Despite advances during the last decade, biological mechanisms underlying NSSI are still not well understood [13].

NSSI is typically performed to inflict harm and/or pain to the surface of the skin, for example by cutting, scratching, and burning skin [14]. Consequently, pain perception has gained attention in the research field of NSSI and individuals with NSSI typically report analgesia and relief after NSSI [15]. In fact, there is some evidence indicating that NSSI is associated with decreased pain sensitivity [16, 17], which has been put forward as a risk factor for severe NSSI [18, 19]. Mürner-Lavancy and colleagues [20] show decreased pain sensitivity to be a possible predictor of NSSI. Further, research on NSSI finds an increased cortisol secretion in response to pain [17] and that an increase in pain-related cortisol secretion varies as a function of NSSI severity, i.e., increased NSSI severity predicts an increased cortisol response in adolescents [21]. There are, however, some conflicting results on the autonomic response to pain in NSSI; one study shows a decreased sympathetic response and another an increased sympathetic response to induced pain [17, 21]. There is also some evidence pointing to childhood adversity, which is also a risk factor for NSSI, influencing the psychophysiological pain response with increased cortisol and autonomic response [22].

In this study, we examined affective touch, a type of tactile stimulus at the opposite end of the affective valence spectrum from pain. This type of touch is conveyed by slowly conducting unmyelinated low-threshold mechanoreceptors (C-LTMRs, often called C-tactile afferents or CTs in humans; [23]). These unmyelinated afferents are poorly suited for transmitting specific information about the discriminative features of touch, such as location and force, which are typically conveyed by large, myelinated afferents, but they encode its affective valence. A critical feature of CTs is that their firing frequency is speed dependent, and correlates with subjective perceived pleasantness for touch, where participants typically rate mid-slow velocities (between 1 – 10 cm/s) as most pleasant [24]. This represents how parents typically touch their children, and how romantic couples touch each other [25–27]. Because CT-mediated touch carries actionable information about safety and affective valence that supports internal bodily awareness, affective touch, like pain, is considered interoceptive in nature [28, 29]. The insular cortex plays a central role in interoceptive integration [30–32], making it particularly relevant for the present study, given both the nature of the tactile stimulus and the clinical characteristics of the NSSI group. In our previous work, we find greater insular activity in individuals with NSSI compared to controls during exposure to affective images [33]. To examine whether similar group differences would emerge for touch processing, we conducted a small-volume correction analysis restricted to the bilateral insula in addition to a traditional whole-brain analysis.

Studies on affective touch and CT fibers in adolescents are scarce, and evidence is less clear. However, CT-targeted tactile stimulation in children and adolescent participants [34, 35] consistently show that slow touch (3 cm/s), is perceived as more pleasant compared to fast touch (30 cm/s). Croy and colleagues [34] also find an age effect, indicating that older children rate affective touch as more pleasant compared to younger children. The study also shows a positive correlation between age and affective touch index, a score indicating an individual’s specific preference for slow over fast touch. Sehlstedt and colleagues [35] find that overall hedonic value of touch increases with age, while the discriminative aspect of touch decreases.

Adolescents with NSSI consistently report more adversities and trauma symptoms than those without NSSI [36, 37]. Such negative experiences can affect experiences of affective and interpersonal touch [38] with CT optimal 3 cm/s touch, typically perceived as pleasant, rated as less rewarding in young adults with negative traumatic childhood experiences compared to controls [39]. Further support for altered affective touch processing in mental disorders was found in a recent review [40]. Participants with psychiatric diagnoses [41], such as borderline personality disorders (BPD; [42]), anorexia nervosa [43] and skin picking disorder [44] generally report less pleasure from affective pleasant touch than controls. Patients with BPD, in which NSSI is a symptom criterion, not only perceived being stroked with pleasant objects as less plesant than controls but also rated painful objects as more pleasant [45].

However, research on clinical groups of adolescents is limited. Research on CT-touch and neural mechanisms of touch processing in adolescents with autism, for example, show an altered relationship between subjective perception and central representation of touch hedonics compared to typically developing adolescents. More specifically, adolescents with autism have marginally lower affective touch awareness and altered neural coupling between right pSTS and touch hedonics, despite there being no differences in their subjective ratings of touch pleasantness compared to controls [46], and reduced activity in brain regions involved in social-emotional and sensory information processing in response to CT-targeted touch [47]. However, to our knowledge, affective touch processing is yet unexplored in NSSI.

There are some recent studies with preliminary evidence of interoceptive deficits in individuals with NSSI [48–53]. Using resting state fMRI, Ho and colleagues [50] found altered activity in networks associated to self-referential and interoceptive processing in NSSI. Specifically, they found lower within-network connectivity in default mode network and in the insula subdivision of the salience network in depressed adolescents with NSSI compared to depressed adolescents without NSSI and controls. However, less is known about interoception in adolescent NSSI, especially in transdiagnostic samples. This is an interesting avenue to explore further, as disconnections from internal body experiences can potentially have negative consequences for self-protective behaviors [49].

Here, we build on the nascent research that focuses on biological mechanisms in NSSI by focusing on a yet unexplored stimulus with interoceptive characteristics, i.e., affective touch. Using both functional magnetic resonance imaging and behavioral touch perception, we aimed to examine how adolescents with NSSI respond to affective touch. Our hypothesis was that adolescents with NSSI would have different subjective perceptions and neural mechanisms of affective and social touch compared to healthy controls.

## Methods

### Participants and procedure

A total of 30 clinical participants and 30 healthy controls were recruited from June 2016 to March 2018. The 30 clinical participants were recruited via the child and adolescent psychiatric clinic in Linköping, Region Östergötland, Sweden. Inclusion criteria for the clinical participants were NSSI, independent of psychiatric diagnosis, having engaged in NSSI at least on five different instances in the last six months, to include those with more repetitive and recent NSSI, and being a female between the ages 15 and 18 years. Exclusion criteria were IQ below 80, diagnosis of schizophrenia, psychotic or bipolar disorders and/or alcohol/drug abuse or dependence. Participants taking psychotropic medications were included if those were unchanged for at least three months. The 30 healthy controls were recruited via advertising at schools and social media. Inclusion criteria for the healthy controls were being a female between the ages 15 and 18, and exclusion criteria were DSM Axis I or II disorder during the last year and any past or present NSSI. There was no statistically significant difference in age, handedness, or parents’ country of origin between the participants in the two groups. There were significant differences between groups for IQ (*p* = 0.03) and family structure (*p* = 0.04), and a trend for parents’ level of education (*p* = 0.045). For participant demographics see Table 1. The sample size was based on previous recent neuroimaging studies on the same clinical population and depended on recruitment, eligibility criteria and by completion of the experimental session. We provide optimal sample size calculated using G*Power 24 [54] by entering a “minimum theoretically informative effect size”, based on effect-size estimates for common fMRI experimental designs (see Box 2 in [55]). Given a 2 × 2 factorial within-between subject design and assuming an effect size of Cohen’s *f* = 0.2, and an *α* = 0.05, a total sample size of 52 subjects is required to detect an effect with ≥ 80% power.

**Table 1:** Participant demographics.

Demographic characteristics	NSSI *N* = 27 *N* (%)	Healthy controls *N* = 26 *N* (%)	Comparison statistic *p*
Sex			
Female	27 (100)	26 (100)	
Age			
*M (SD)*	16.0 (0.8)	16.3 (0.9)	0.16
IQ			
*M (SD)*	95.9 (9.7)	102.1 (10.4)	0.03
Handedness			
*M (SD)*	78.8 (28.8)	76.2 (45.9)	0.56
Parental education (NSSI *N* = 50, controls *N* = 45)			
University/college	21 (42.0)	29 (64.4)	0.045
Theoretical high-school program	3 (6.0)	5 (11.1)	
Vocational high-school program	22 (44.0)	10 (22.2)	
Compulsory school	4 (8.0)	1 (2.2)	
Parent born in other country (NSSI *N* = 52, controls *N* = 48)	3 (5.8)	4 (8.3)	0.60
Current family structure⁑			
Married/co-habitant	11 (40.7)	17 (68.0)	0.04
Divorced	16 (59.3)	7 (28.0)	
Single parent household	0 (0)	1 (4.0)	
NSSI			
Age of onset *M (SD)*	13.2 (1.3)		
Number of methods *M (SD)*	3.7 (1.9)		
Past year cutting frequency⁑ *M (SD), median*	67.9 (87.0) 30.0		
Psychiatric diagnoses*			
Depression	13 (48.1%)		
Anxiety disorder	12 (44.4%)		
Posttraumatic stress disorder	1 (3.7%)		
Borderline traits	11 (40.7%)		
Eating disorder	6 (22.2%)		
ADHD/ADD	13 (48.1%)		
High functioning autism	4 (14.8%)		
ODD/CD	3 (11.1%)		
Medications **			
SSRI/SNRI	7 (25.9)		
SSRI/SNRI + Methylphenidate	1 (3.7)		
Neuroleptic	1 (3.7)		
SSRI/SNRI + Neuroleptic	1 (3.7)		
No medication	17 (63.0)		

⁑missing data for one participant.

*each participant could have several diagnoses.

**medications at time of fMRI.

The study and all procedures were approved by the Regional Ethical Board of Linköping (Dnr 2015/273-31; 2016/224-32). All participants in this study received both oral and written information about the study and all the participants and their caregivers gave written informed consent. Participants were assessed for psychiatric diagnoses by a clinician with extensive experience of child and adolescent psychiatry. On another day, participants were introduced to the MR-environment in a mock scanner (see supplement), which was followed by a session in the magnetic resonance imaging (MRI) scanner at the Center for Medical Image Science and Visualization (CMIV), Linköping. During this session, behavioral and fMRI data on touch processing were collected. Participants with complete touch data were included in the current study, resulting in a final sample for analysis consisting of 27 participants with NSSI and 26 controls.

### Clinical assessment

Demographic questions were used for collecting data on gender, age, living situation, and parents’ education and immigrant background through self-reporting in fixed answer categories (Table 1). Before the experimental part of the study, participants went through clinical assessments: Clinician-Administered NSSI Disorder Index (CANDI; [56]) to assess information on NSSI, and The Schedule for Affective Disorders and Schizophrenia for School-Age Children – Present and Lifetime version (K-SADS-PL; [57]) to assess psychiatric diagnoses. To assess cognitive functioning, the abbreviated versions of Wechsler Intelligence Scales, fourth edition for children [58] or adults [59] were used, depending on participants’ ages.

### Self-report

The Social Touch Questionnaire (STQ; [60]) was used to assess individuals’ perception about social touch. The questionnaire consists of 20 items covering attitudes towards social touch, i.e., both appreciation of touching and being touched. Items are rated on a five-grade Likert scale from strongly disagree to strongly agree, and a high score indicates an aversion towards social touch. The Trauma Symptom Checklist for Children (TSCC; [61]) was used to evaluate possible symptoms and responses to traumatic events. TSCC consists of 54 items, which are rated on a four-point Likert scale from never to almost always, with high scores indicating greater symptomatology.

### Task

Participants engaged in three tasks in the MRI scanner, which included a social processing task [12], an affective picture processing task [33], and a tactile stimulation task, which is the focus of the current study. As participants lied in the MRI scanner, they received sequences of slow and fast tactile stimulation in a counterbalanced order, as previously reported [46]. Brush strokes were manually delivered over 9 cm on the dorsal part of the right forearm at two different velocities, at CT-optimal speed (slow touch, ~ 3 cm/s) and at non-CT-optimal speed (fast touch, ~ 30 cm/s). Each stimulation lasted for 12 s, was delivered by a trained experimenter guided by an audio script, and was followed by a jittered interstimulus interval of 10–12 s. Stimulation trials were presented 5 times per velocity and in three consecutive runs, for a total of 15 trials per velocity. Participants were asked to rate using a button pad positioned in their left hand, pleasantness and intensity of the stimuli on a visual analog scale (VAS) displayed for 6 s, with the scale anchors “unpleasant-pleasant” and “not intense-intense”, 3 times in total per velocity. The scale was later converted to the range −10– + 10 for calculating the pleasantness and intensity ratings. In addition, the “affective touch index” score, which reflects the extent of discrimination between CT-optimal (slow) and non-CT-optimal (fast) brushing speeds, was calculated and compared between groups using an independent samples t-test. Affective touch index scores were converted to the range 0–10, as in Croy et al. [26]. Previous research used either the “affective touch index” or the “affective touch awareness score” to investigate this discrimination ability. The affective touch index is calculated as the difference in pleasantness ratings for slow and fast, divided by overall within-subject pleasantness ratings, while the affective touch awareness score is calculated as the difference in pleasantness ratings for slow and fast touch multiplied by overall within-subject pleasantness ratings. Three articles have used the affective touch index [34, 62, 63], while two articles have used the affective touch awareness score [26, 46].

### MRI data preprocessing and analysis

MRI data acquisition details are described in the supplement. Data analysis was performed with the Analysis of Functional Neuro Images (AFNI) software, version 23.1.02 [64, 65], using recommended specifications from example 6b on the afni_proc.py documentation. EPI images were slice-time corrected, smoothed with a kernel size of 4 mm, registered to minimum outlier volume, and transformed to Montreal Neurological Institute (MNI) space using non-linear registration, using the output from the @SSWarper function. Motion parameters and their derivatives associated to head motion were added as regressors of no interest. A motion censoring threshold of 0.3 mm per TR and an outlier fraction threshold of 0.05 were used, and volumes exceeding these values were not included in the time-series regression (see supplement Table S1 for TR censored per subject). Voxel-wise general linear model (GLM) statistical analysis was carried out on the BOLD time-series data using the 3dDeconvolve function. Two boxcar regressors were created, one for slow and one for fast stimulation epochs. In addition, two boxcar regressors modeling pleasantness and intensity rating epochs, which included motor presses, were created. For group analysis, we used two statistical approaches. We previously observed in NSSI versus controls, higher activity in the insular cortex during exposure to affective images [33]. In addition, insula is a region of interoceptive integration [30–32], making it a relevant region in terms of the type of stimulus and the clinical group investigated here. To assess whether the two groups were different in this region during touch, we performed a small-volume correction analysis focusing on bilateral insula taken from the Eickhoff-Zilles macro labels from N27 in MNI space (CA_ML_18_MNI atlas in AFNI). In addition, to assess general brain activity during touch, and potential between-group differences, we performed a whole-brain analysis.

For both approaches, a 2 × 2 factorial ANOVA with “speed” as a within-subject factor (levels: slow, fast) and “group” as a between subject factor (levels: NSSI, control) was performed using the AFNI function 3dMVM [66]. Three general linear tests were included in the 3dMVM syntax, to address the directionality of the speed and group effects (i.e. slow vs fast), and to compare slow and fast conditions to baseline (i.e. slow vs baseline, fast versus baseline). Results were thresholded at a per-voxel *p* = 0.002, cluster corrected at *alpha* = 0.05 [67]. To achieve multiple comparison corrected results, spatial group smoothness parameters estimated from the residuals were used in a 3dClustSim -acf simulation of bilateral insula mask for the small-volume approach, and a grey matter group mask for the whole-brain approach. The gray matter mask was based on the union of EPI-masks of subjects surviving the different types of censoring, multiplied with an MNI grey matter mask to reduce the number of voxels used for statistical testing to voxels lying in grey matter.

No additional analyses addressing potential associations between behavioral ratings and brain activity were performed as those require large sample sizes to be considered meaningful [68].

### Behavioral data analysis

Data were analyzed with descriptive statistics using frequencies, percentages, mean values and standard deviations, and cross-tabulation with Fisher’s exact test for categorical data. Independent samples t-test for group comparisons was used with Cohen’s *d* for effect size (ES) with 0.2, 0.5 and 0.8, indicating small, medium and large effects [69]. Data were normally distributed (Shapiro Wilks’ ps > 0.2). Average pleasantness and intensity touch ratings per participant were entered in a repeated measures ANOVA with “speed” as within-subject factor (levels: slow, fast) and “group” as between subject factor (levels: NSSI, control) with partial eta squared (*η²p)* for ES with 0.01, 0.06, and 0.14 indicating small, medium and large effect. To explore the potential effect of trauma, we performed an additional 2 × 2 ANCOVA including TSCC total scores as covariate. The affective touch index was calculated as the difference in pleasantness ratings for slow and fast, divided by the average of all within-subject pleasantness ratings [34, 62, 63]. Post hoc tests were performed using t-tests. Statistical analyses were performed using IBM’s Statistical Package for the Social Sciences (SPSS) version 29.0 and figures were made using GraphPad Prism, version 10.

## Results

### Pleasantness and intensity ratings

**Fig. 1:**
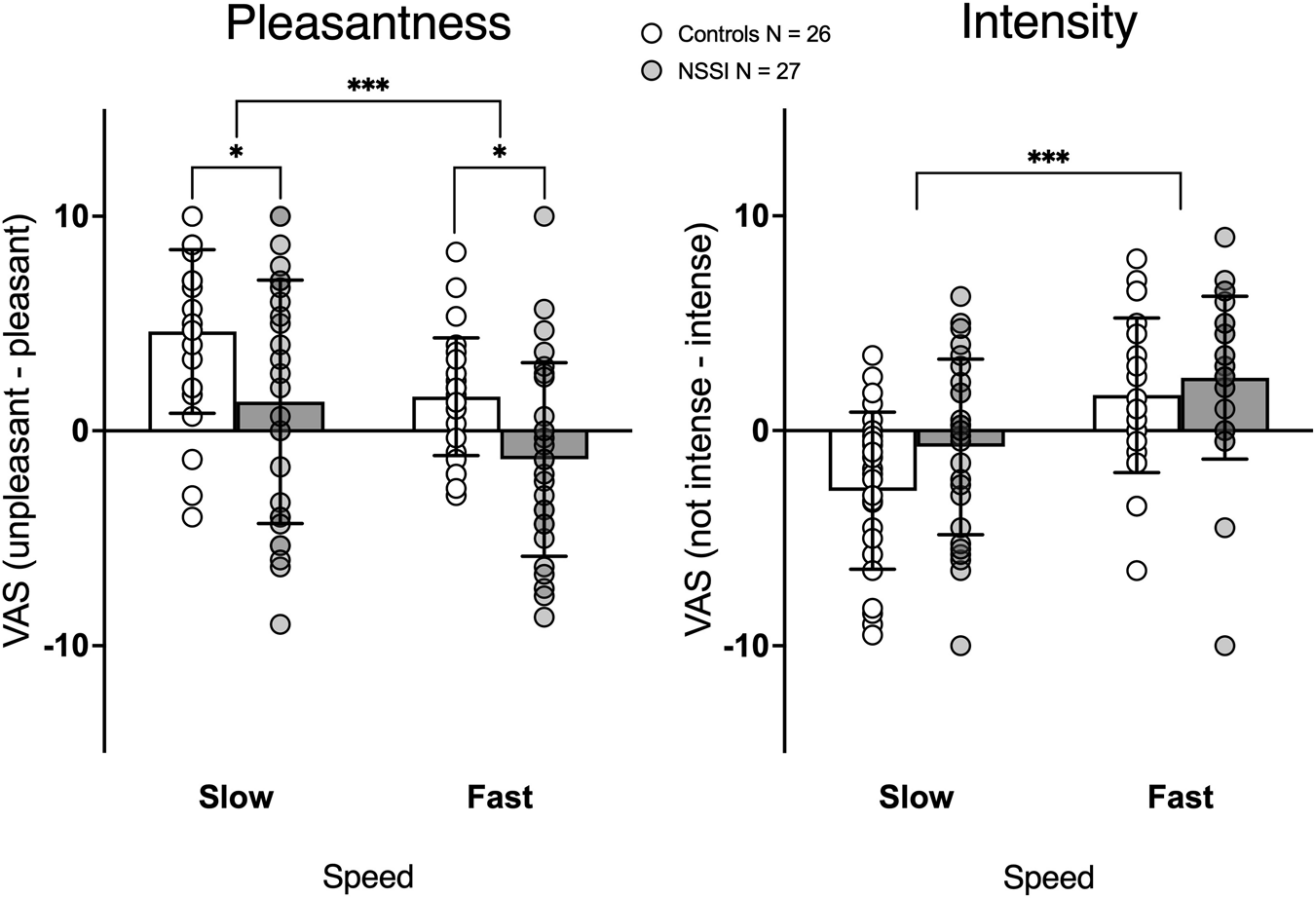
Behavioral results. (**Left**) Pleasantness ratings. Bar charts depicting pleasantness ratings associated to slow and fast brushing. Slow brushing was perceived as more pleasant than fast brushing in both groups. Compared to controls, the NSSI group had lower pleasantness scores for both slow and fast touch. (**Right**) Intensity ratings. Fast brushing was perceived as more intense than slow brushing in both groups. * *p* = 0.05, *** *p* < 0.001. Error bars indicate standard deviation of the mean (SD).

For pleasantness ratings, a significant main effect of speed was observed [Fig. 1; *N* = 26 Control, *N* = 27 NSSI; *F* (1, 51) = 23.3, *p* < 0.001, *η²p* = 0.31], with slow touch rated as more pleasant than fast touch (*t* = 4.87, *p* < 0.001; Control_slow_ = 4.63 ± SD 3.81; NSSI_slow_ = 1.36 ± SD 5.66; Control_fast_ = 1.59 ± SD 2.74; NSSI_fast_ = −1.33 ± SD 4.50). A main effect of group was also observed [*F* (1,51) = 8.98, *p* = 0.004, *η²p* = 0.15]. Compared to controls, the NSSI group had lower pleasantness ratings, both for slow and fast touch (*t*_*slow*_ = 2.46, *p* = 0.017, *t*_*fast*_ = 2.83, *p* = 0.007). No speed by group interaction was observed (*p* > 0.8). Given our transdiagnostic NSSI sample, individual data points for participants with comorbid diagnosis are presented in Fig. S1. Further, exclusion of participants with autism (*N* = 4) and eating-disorders (*N* = 6) still showed a speed and group effect (see supplement). IQ differed between groups but did not significantly correlate with pleasantness ratings (*ps* > 0.4), violating the purpose of ANCOVA. Therefore, we report the results without IQ as a covariate.

TSCC total scores correlated negatively with pleasantness ratings (*r* = −0.38, *p* = 0.006 for slow, *r* = −0.41, *p* = 0.003 for fast) but showed no significant association with intensity ratings (*ps* > 0.06). We then performed an ANCOVA, with speed and group as factors and TSCC as covariate on pleasantness ratings only. Significant main effects were observed for TSCC (*F*(1,51) = 4.41, *p* = 0.041, *η²p* = 0.08) and speed (*F*(1,51) = 4.52, *p* = 0.039, *η²p* = 0.08). The main effect of group was no longer significant (*p* = 0.99). For the affective touch index score, no significant difference between the two groups was identified (*t* = - 0.29, *p* = 0.77). For intensity ratings, a significant main effect of speed was observed [*N* = 26 Control, *N* = 27 NSSI; *F* (1,51) = 47.17, *p* < 0.001, *η²p* = 0.48], with fast touch rated as more intense than slow touch (*t* = 6.83, *p* < 0.001; Control_slow_ = −2.79 ± SD 3.65; NSSI_slow_ = −0.75 ± SD 4.08; Control_fast_ = 1.65 ± SD 3.59; NSSI_fast_ = 2.46 ± SD 3.80). No group effect or speed by group interaction were observed (*p*_*s*_ > 0.3).

### fMRI

One NSSI participant was not included in the MRI data analysis for issues with the raw MRI data, resulting in *N* = 26 NSSI and *N* = 26 controls included in the MRI analysis.

**Fig. 2:**
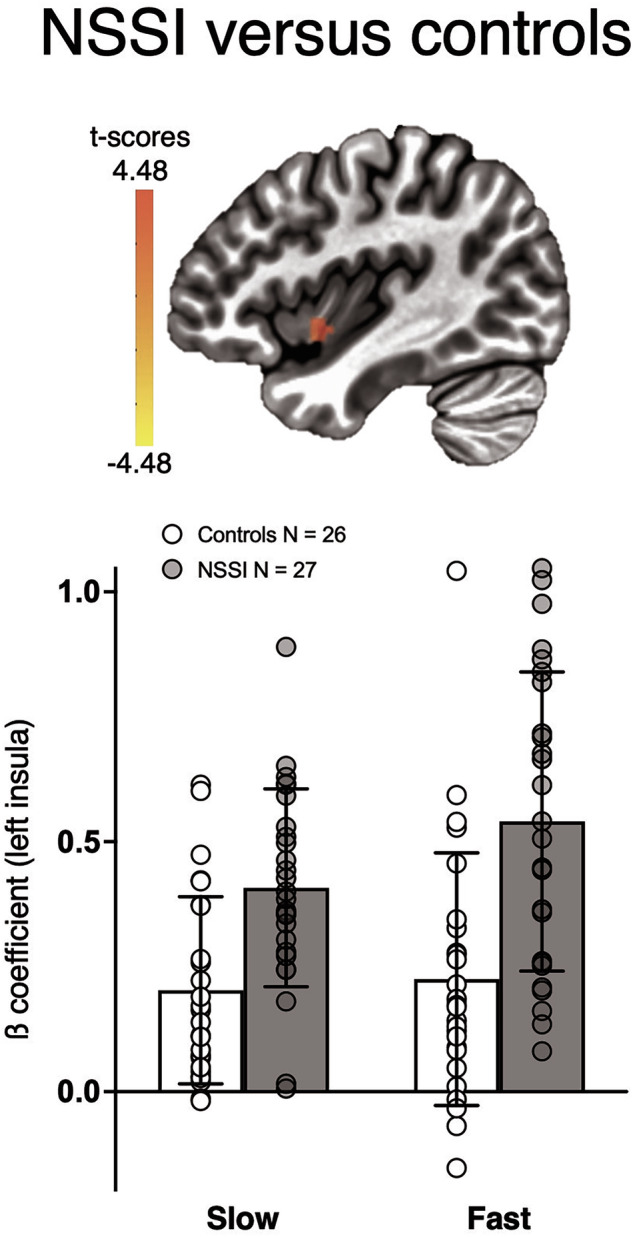
Insula fMRI results. Activation map showing the main effect of group in left insula (MNI = −41, 1, −8, cluster size = 9), with directionality related to the contrast NSSI > controls (*p* < 0.002, small-volume, bilateral insula mask cluster corrected at *alpha* = 0.05). Bar-chart showing average ß coefficients for each participant for slow and fast speeds in left insula. The insula mask was created using the Eickhoff-Zilles macro labels from N27 in MNI space (CA_ML_18_MNI atlas in AFNI).

For the small-volume corrected analysis focusing on bilateral insula, a main effect of group was found in left insula (MNI = −41, 1, −8, cluster size = 9), with significantly higher ß coefficient values in NSSI compared to controls (Fig. 2). This difference was independent of brushing speed.

**Fig. 3:**
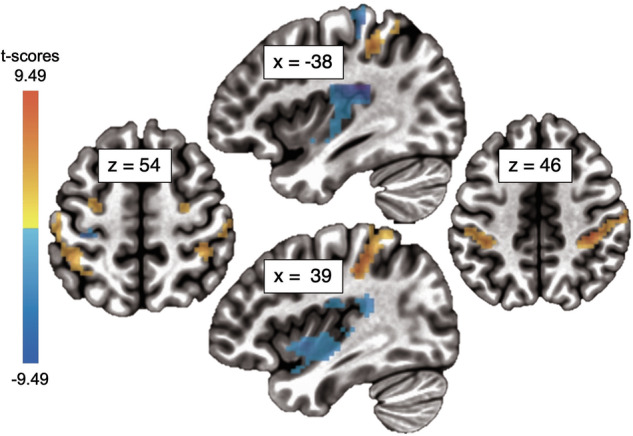
Whole-brain fMRI results. Activation map showing the main effect of speed, with directionality related to the contrast slow > fast (*p* < 0.002, whole-brain, grey matter, cluster corrected at *alpha* = 0. 05).

For the whole-brain analysis, an effect of speed was found (Table 2, Fig. 3) but no effect of group or group x speed interaction. We identified two clusters in the primary somatosensory cortex differentially involved in slow and fast touch, as previously reported in a different adolescent population [46]. A more rostral cluster was active to fast versus slow, whereas a more caudal cluster was identified for the opposite contrast. Specifically, compared to slow touch, fast touch resulted in higher ß coefficients in left primary somatosensory cortex, contralateral to tactile stimulation (Area 1-3b), whereas the opposite contrast showed increased activation bilaterally in Area 2. In addition, the fast versus slow touch contrast showed activity in left secondary somatosensory, merging with posterior insula. Additional regions for the same contrast included bilateral hippocampus and right mid-to-posterior insula.

**Table 2:** Activations associated with 2 × 2 ANOVA whole-brain analysis, expressed by peak scores in *Montreal* Neurological Institute (MNI) space coordinates (x, y, z).

Analysis	Region	MNI coordinates (x, y, z)			Voxel #
Slow > Fast					
	Right_Postcentral_Gyrus (Area 2)	37	−32	46	257
	Left_Postcentral_Gyrus (Area 2)	−38	−38	46	197
	Left_Precentral_Gyrus (Area 6)	−23	−11	67	49
	Right_Precentral_Gyrus (Area 6)	28	−11	61	47
Fast > Slow					
	Right_Fusiform_Gyrus	34	−47	1	638
	Left_Rolandic_Operculum	−38	−29	22	556
	L_Hippocampus	−20	−32	−8	181
	R_Thalamus	7	−2	−5	122
	Left_Cerebellum_(IX)	−14	−47	−41	121
	Left_Superior_Occipital_Gyrus	−17	−86	7	102
	Right_Calcarine_Gyrus	25	−71	10	76
	Left_Postcentral_Gyrus (Area 1)	−35	−32	64	61
Slow > Baseline					
	Left_Postcentral_Gyrus	−62	−20	34	11491
	Left_Middle_Occipital_Gyrus*	−29	−89	1	3435
	Left_Superior_Frontal_Gyrus	−11	22	43	261
	Right_Precentral_Gyrus*	22	−23	73	189
	Left_Precentral/Postcentral_Gyrus*	−41	−20	64	185
	Left_Precentral_Gyrus (Area 6)	−23	−14	67	88
	Left_Middle_Temporal_Gyrus	−56	−71	10	63
Fast > Baseline					
	Left_Rolandic_Operculum	−38	−20	19	14664
	Left _Middle_Occipital_Gyrus*	−32	−77	25	2343
	Right_Precentral_Gyrus (Area 6)*	31	−14	70	249
	Left_Postcentral_Gyrus*	−44	−20	61	125
	Left_Posterior_Cingulate	−8	−29	46	50

Per-voxel *p* < 0.002, cluster corrected at *alpha* = 0.05. *indicate regions with negative t-scores.

**Fig. 4:**
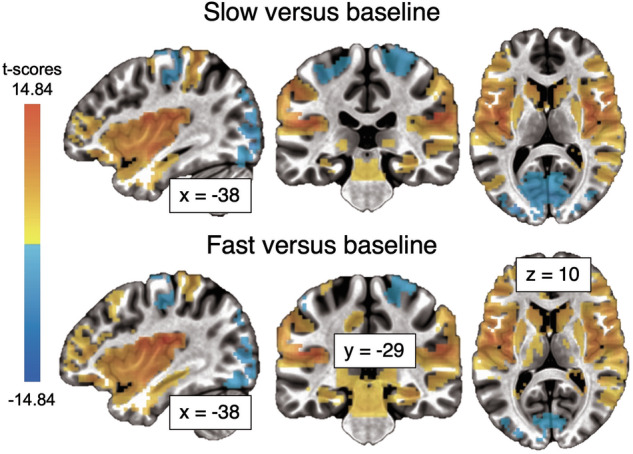
Whole-brain fMRI results. Activation map showing the contrasts slow > baseline and fast > baseline (*p* < 0.002, whole-brain, grey matter, cluster corrected at *alpha* = 0.05).

When compared to baseline, large clusters for the slow and fast speeds were identified (Table 2, Fig. 4). The clusters included widespread activity in some of the typical regions involved in tactile processing, including bilateral primary and secondary somatosensory cortices, bilateral insula, and right superior temporal sulcus, ipsilateral to stimulation side (Table 2, Fig. 4). In addition, bilateral activity in dorsolateral prefrontal cortex and in subcortical regions including caudate, putamen and amygdala were identified. Negative ß coefficients were identified bilaterally in primary motor cortex and visual regions, including primary and secondary visual cortex in the occipital lobe, and more dorsally and rostrally in the parietal cortex.

### Self-reported perception of social touch and trauma symptoms

**Fig. 5:**
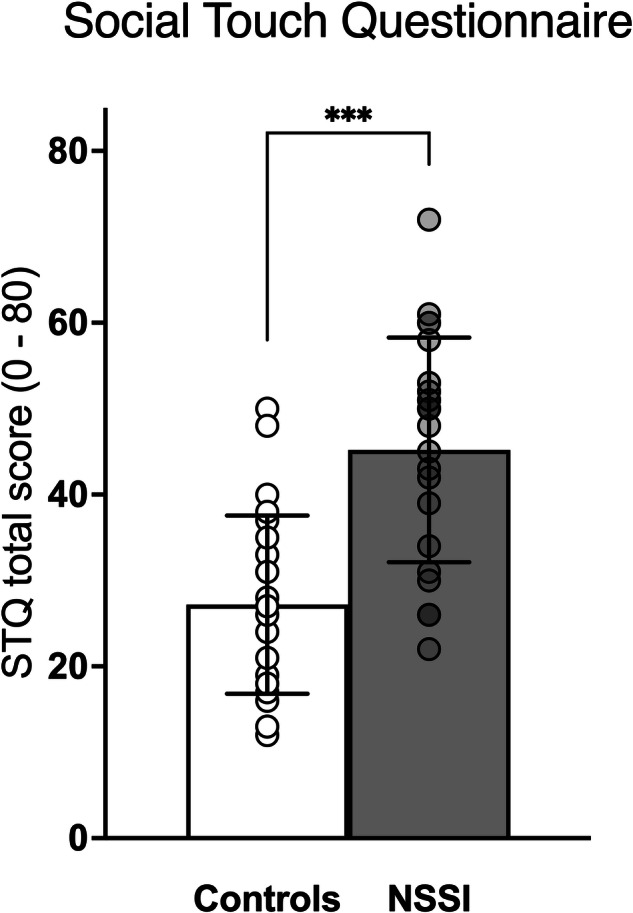
Social Touch Questionnaire results. Bar charts depicting STQ scores. Social touch was perceived as more aversive in NSSI compared to controls. *** *p* < 0.001. Error bars indicate standard deviation of the mean (SD).

The NSSI group had significantly higher scores, *M* = 45.2 (*SD* = 13.1), compared to controls, *M* = 27.2 (*SD* = 10.4), on the STQ (*N* = 26 Control, *N* = 25 NSSI, *t* = 5.46, *p* < 0.001, Cohen’s *d* = 1.53), indicating a stronger avoidance of social touch in the NSSI group with a large effect size (Fig. 5). The NSSI group also reported higher scores on the TSCC, *M* = 63.2 (*SD* = 21.2), compared to controls *M* = 20.0 (*SD* = 10.9), indicating higher levels of trauma symptoms (*N* = 26 Control, *N* = 25 NSSI, *t* = 9.11, *p* < 0.001, Cohen’s *d* = 2.58) with a large effect. Some missing data on self-report questionnaires for two participants in the NSSI group resulted in *N* = 25 for these analyses.

## Discussion

This study provides the first evidence of altered tactile processing in NSSI, with reduced pleasantness for touch in adolescents with NSSI versus controls. In addition, participants with NSSI rated higher avoidance of social touch. We further found increased insula activity to touch in the NSSI group compared to controls, but no between-group differences in the whole-brain analysis. The perception of discriminative aspects of touch was similar between NSSI and controls.

We found that the affective but not the discriminative aspects of touch were perceived differently in NSSI compared to controls. Our findings showed that although individuals with NSSI demonstrated good discrimination between slow and fast touch, their overall pleasantness ratings were lower compared to the control group. Specifically, we replicated the expected pattern in both groups: slow touch was rated as more pleasant and less intense than fast touch. This suggests that both groups could similarly differentiate between slow and fast tactile stimuli, though individuals with NSSI found touch, in general, to be less pleasant. While pain has been more extensively studied in adolescents with NSSI [16, 17, 21], both pain and affective touch represent interoceptive modalities, and our findings suggest that alterations may extend beyond nociception to other forms of interoceptive processing.

We found that self-reported trauma symptoms contributed to altered pleasantness for touch. Since TSCC scores differed significantly between groups, the covariate was not statistically independent of the group factor. Therefore, these results cannot establish that trauma symptoms fully account for the group effect but suggest that they may contribute to the observed group differences in pleasantness ratings. This finding confirms earlier studies showing that individuals with trauma experiences have altered perception of affective touch [38–40].

There was no significant difference in the affective touch index score between the two groups, likely because the relative difference in pleasantness ratings within the NSSI group was comparable to that of the control group. These findings are consistent with prior research on affective touch in healthy participants, both adults and adolescents, which indicates that slow touch is perceived as more pleasant [24, 34].

Our finding of altered perception of affective touch in a transdiagnostic sample of adolescents with NSSI is also found in adult psychiatric populations [40]. However, despite responses to touch being shared across different psychiatric groups, some responses are not, and Papi and colleagues raise the question whether these alterations in somatosensory processing are disorder-specific or an expression of an underlying transdiagnostic trait.

We also found a difference between groups in self-reported perceptions of social touch. The higher scores in the NSSI group indicated more aversion against social touch, which possibly could be a part of why they also report lower pleasantness to touch. To our knowledge, there are no previous studies using the STQ for adolescents with NSSI. As a reference, the STQ scores in the current study were higher in our transdiagnostic female sample with NSSI compared to both female undergraduate students with high levels of social anxiety [60], a healthy student sample [70], and also an adolescent sample (85% male) with autism [46].

At the brain level, we observed greater insula activity to slow and fast touch in the NSSI group compared to controls. This aligns with our previous study, which by combining neuroimaging and psychophysiological data found that insula activity in NSSI patients was positively correlated with facial EMG scores, a marker of heightened emotional reactivity, to both positive and negative affective images, an effect not observed in controls [33]. Two key points underscore the significance of these findings. First, both studies demonstrate heightened insula activity in the NSSI group, although in different insular regions: the anterior insula in the previous study and the mid-insula in the present study. Second, the previous study shows that NSSI patients exhibit heightened emotional reactivity to both positive and negative visual stimuli [33]. This general reactivity aligns with our current finding of generally increased insula activation to touch in NSSI, as heightened activity was observed in response to both slow and fast touch, stimuli usually perceived as having different hedonic values. Together, these studies suggest that heightened insula activation in NSSI might reflect enhanced reactivity to affective cues, highlighting differences in interoceptive processing in this population.

Except for the observed difference in insula activity, no between-group differences were identified in the whole-brain analysis. We found similar neural activation in NSSI and controls to CT-optimal and CT-non-optimal touch, with activations in primary and secondary somatosensory cortices and posterior insula to both fast and slow touch, replicating previous findings [71–73]. When comparing the two speeds, we found that distinct regions within the primary somatosensory cortex responded differentially to slow and fast touch, as we reported in an earlier study in male adolescents [46]. Specifically, fast touch activated a more rostral cluster in the left primary somatosensory cortex (Area 1-3b, contralateral to stimulation), whereas slow touch elicited greater bilateral activation in a more caudal cluster (Area 2). This pattern suggests that the adolescent somatosensory cortex might process tactile stimuli with varying hedonic and sensory properties through distinct spatially organized areas.

In this and our previous study [46], we did not observe posterior insula activation for the slow versus fast touch contrast. Instead, here we found that the posterior insula was more engaged during fast touch than slow touch, which is inconsistent with prior findings in adults showing greater posterior insula activation for slow, CT-optimal touch [73]. This discrepancy could be due to several factors discussed below.

First, our study population consisted of adolescents, while most prior research has focused on adults, so developmental differences may contribute to the observed variation in insular response. Second, publication bias may play a role, as studies that do not replicate the expected pattern of greater activation for slow touch in the posterior insula may be underreported. In addition, recent evidence suggests that the insular cortex can respond to both CT-optimal and CT-non-optimal touch [71, 72, 74, 75], which challenges the notion of the posterior insula as selective cortical target for CT-optimal, affective touch. These findings point to a need for further research with larger sample sizes and a broader age range to better understand the role of the posterior insula across different touch modalities and developmental stages.

This study has some limitations, one being the relatively small sample size, which excluded some further subgroup analysis. In this study, the participants were brushed manually by the experimenters, compared to other studies that have used a mechanical device to deliver the brush strokes (e.g., [24, 34]). One study by Triscoli and colleagues [76] investigated whether there is a difference in pleasantness ratings when using a mechanical device to deliver tactile stimuli compared to if the tactile stimuli are delivered manually by a person. This study, performed in healthy participants, found that there is no significant difference in pleasantness ratings when comparing the two methods. Despite these results, it cannot be excluded that human-delivered versus machine-delivered touch might have triggered a different perception in our sensitive population of NSSI adolescents, even if the participants could not see the experimenters during the task. In clinical samples, NSSI is overrepresented in females, and our sample was all female, which limits generalization to all adolescents with NSSI. We do not have data on hormonal status and menarche for all participants, which could potentially influence results. It has recently been shown that adult females in luteal phase showed higher peripheral oxytocin release during self- and other-touch than females in follicular phase. However, pleasantness ratings did not differ between phases [77]. Finally, an important limitation to consider is the lack of a clinical control group without NSSI. The transdiagnostic sample used in this study is a strength but has limitations. The sample had several comorbidities, such as depression and different neurodiversities, including attention deficit hyperactivity disorder (ADHD) and high-functioning autism. Lacking a clinical control group could potentially impact our conclusions. Altered perception of social touch is a common feature in autism, for example [46], and self-reported anxiety from social touch has also been found in individuals with high levels of social anxiety [60]. Levels of social anxiety, which could contribute to social-touch aversion and affective-touch pleasantness ratings in adolescents, were not measured in the current sample, which is a limitation. With the current design and analysis, we cannot say for certain that the differences found are due to NSSI specifically, or to one or several comorbidities. However, several different diagnoses were represented, and we performed subgroup analyses and covariate analysis of trauma symptoms to control for effects. The NSSI group had higher levels of self-rated trauma symptoms. Childhood adversities influence reactions to pain [22], and it is therefore not unlikely that this would also influence reactions to affective touch [38]. We have no way of making causal inferences with the current design, and the exact mechanisms involved in the alteration in affective touch need to be examined further.

### Future research and clinical implications

Affective touch has the potential to reduce stress and pain. If social touch is perceived as less pleasant with altered neural processing, this could influence the effect of social touch, potentially preventing the beneficial effects on stress reduction and social bonding. A blunted response to interoceptive stimuli could further influence self-protective behaviors. Interoceptive processing could therefore be an interesting avenue to examine further in individuals with NSSI to increase clinical attention to this phenomenon. Improving interoceptive processing through training is largely unexplored in the field of NSSI. Further studies are needed on the therapeutic potential of affective touch.

## Supplementary information

Can you feel it? Altered affective touch perception in a transdiagnostic sample of female adolescents with nonsuicidal self-injury Supplement Material: Methods and Results

Figure S1

## Data Availability

Data are not publicly available since we do not have participants’ or ethical permission to share data. Data are, however, available from the corresponding author upon reasonable request subject to ethical permissions and participant consent.
