# Supplementary material for: Can you feel it? Altered affective touch perception in a transdiagnostic sample of female adolescents with nonsuicidal self-injury
Source: Transl Psychiatry. 2025 Nov 24;15:516. doi: 10.1038/s41398-025-03759-9 (PMC12669740; doi:10.1038/s41398-025-03759-9)
Supplement: Supplementary file 1 — Can you feel it? Altered affective touch perception in a transdiagnostic sample of female adolescents with nonsuicidal self-injury Supplement Material: Methods and Results [file 41398_2025_3759_MOESM1_ESM.docx]

**Can you feel it? Altered affective touch perception in a transdiagnostic sample of female adolescents with nonsuicidal self-injury**

**Supplement Material: Methods and Results**

**Methods**

**Mock scanner**

Before the MRI session, participants underwent a training session in an MR simulator (Mag- netic Resonance Simulator (PST MR Simulator System, BlindSight GmbH, Schlitz, Germany). During the training session, participants trained to lie still via feedback from a motion tracking system positioned around their head (MoTrak Head Motion Tracking System, Psychology Software Tools, Sharpsburg, PA, USA), and habituated to the MRI environment. In addition, the participants received instructions and did a trial run of the task. The training session was followed by an MRI session at the Center for Medical Image Science and Visualization (CMIV), Linköping University Hospital.

**MRI Session and Data Acquisition**

Imaging was performed using a Philips Ingenia 3 Tesla MR scanner (Philips Healthcare, Best, The Netherlands) equipped with a 32- channel Philips dS Head head-coil. Blood oxygen-level-dependent (BOLD) data were acquired with echo-planar imaging (EPI) sequences: TR = 2000 ms; TE = 30 ms; flip angle = 77°; field-of-view = 220 × 220 mm^2^; voxel size = 3.4 × 3.4 x 4.0 mm; no slice gap; number of sagittal slices = 32. Three identical functional runs were collected in total. A high-resolution 3D T1-weighted Turbo Field Echo scan was acquired before the EPI data acquisition: TR = 7.0 ms; TE = 3.2 ms; flip angle = 9°; field-of-view = 256 × 240 × 170 mm; voxel size = 1 × 1 × 1 mm; no slice gap; number of sagittal slices = 170.

**Results**

**Subgroup analyses**

We performed two additional analyses where we excluded four individuals with autism and six individuals with eating disorders. The results still showed a speed (*F* (1, 47) = 24.6, *p* < 0.001, *η²p* = 0.34) and a group effect (*F* (1, 47) = 8.4, *p* = 0.006, *η²p = 0.15*) when excluding individuals with autism. The results still showed a speed (*F* (1, 45) = 23.9, *p* < 0.001, *η²p = 0.35*) and a group effect (*F* (1, 45) = 7.5, *p* = 0.009, *η²p* = 0.14) when excluding individuals with eating disorders.

**Figure S1.** Pleasantness ratings. Bar charts showing pleasantness ratings associated to slow and fast brushing in controls. For the NSSI group, six bar charts are shown; individual data points in red indicate participants with a comorbid diagnosis.

**Table S1.** Percentage of removed volumes violating motion censoring threshold of 0.3 mm.

| NSSI (%) | Controls (%) |
| --- | --- |
| 0.2 | 0.0 |
| 4.8 | 3.9 |
| 1.5 | 1.3 |
| 0.2 | 0.2 |
| 0.9 | 0.0 |
| 0.0 | 10.3 |
| 0.0 | 1.5 |
| 0.2 | 14.7 |
| 0.9 | 23.0 |
| 8.6 | 34.6 |
| 3.1 | 10.1 |
| 0.0 | 24.1 |
| 4.8 | 6.8 |
| 8.6 | 0.7 |
| 9.5 | 18.9 |
| 30.9 | 36.4 |
| 1.3 | 3.3 |
| 2.2 | 0.0 |
| 5.7 | 36.8 |
| 0.0 | 0.4 |
| 0.0 | 0.0 |
| 6.1 | 4.2 |
| 0.4 | 0.0 |
| 0.2 | 10.7 |
| 11.0 | 0.0 |
|  | 0.2 |
