# Supplementary figures and images for: Can you feel it? Altered affective touch perception in a transdiagnostic sample of female adolescents with nonsuicidal self-injury
Source: Transl Psychiatry. 2025 Nov 24;15:516. doi: 10.1038/s41398-025-03759-9 (PMC12669740; doi:10.1038/s41398-025-03759-9)

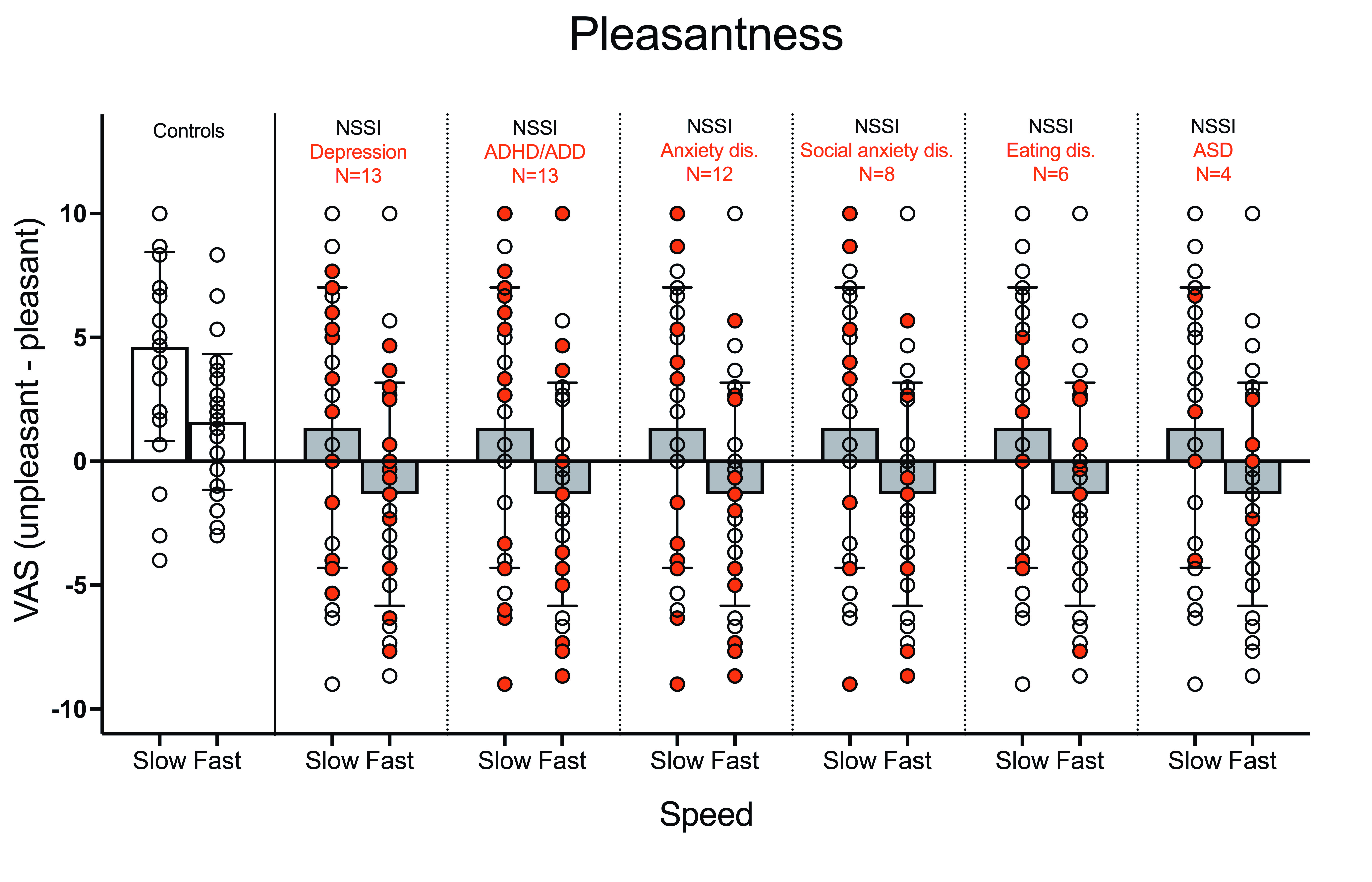

Supplement: Supplementary file 2 — Figure S1 [file 41398_2025_3759_MOESM2_ESM.jpg]
